# Regulation of Copper Homeostasis and Biotic Interactions by MicroRNA 398b in Common Bean

**DOI:** 10.1371/journal.pone.0084416

**Published:** 2014-01-06

**Authors:** Loreto Naya, Sujay Paul, Oswaldo Valdés-López, Ana B. Mendoza-Soto, Bárbara Nova-Franco, Guadalupe Sosa-Valencia, José L. Reyes, Georgina Hernández

**Affiliations:** 1 Centro de Ciencias Genómicas, Universidad Nacional Autónoma de México, Cuernavaca, Morelos. México; 2 Laboratorio de Bioquímica, Facultad de Estudios Superiores Iztacala, Universidad Nacional Autónoma de México. Tlalnepantla, Estado de México. México; 3 Instituto de Biotecnología, Universidad Nacional Autónoma de México, Cuernavaca, Morelos. México; University of Texas, United States of America

## Abstract

MicroRNAs are recognized as important post-transcriptional regulators in plants. Information about the roles of miRNAs in common bean (*Phaseolus vulgaris* L.), an agronomically important legume, is yet scant. The objective of this work was to functionally characterize the conserved miRNA: miR398b and its target Cu/Zn Superoxide Dismutase 1 (*CSD1*) in common bean. We experimentally validated a novel miR398 target: the stress up-regulated *Nodulin 19* (*Nod19*). Expression analysis of miR398b and target genes –*CSD1* and *Nod19*- in bean roots, nodules and leaves, indicated their role in copper (Cu) homeostasis. In bean plants under Cu toxicity miR398b was decreased and *Nod19* and *CSD1*, that participates in reactive oxygen species (ROS) detoxification, were up-regulated. The opposite regulation was observed in Cu deficient bean plants; lower levels of *CSD1* would allow Cu delivery to essential Cu-containing proteins. Composite common bean plants with transgenic roots over-expressing miR398 showed *ca*. 20-fold higher mature miR398b and almost negligible target transcript levels as well as increased anthocyanin content and expression of Cu-stress responsive genes, when subjected to Cu deficiency. The down-regulation of miR398b with the consequent up-regulation of its targets was observed in common bean roots during the oxidative burst resulting from short-time exposure to high Cu. A similar response occurred at early stage of bean roots inoculated with *Rhizobium tropici*, where an increase in ROS was observed. In addition, the miR398b down-regulation and an increase in *CSD1* and *Nod19* were observed in bean leaves challenged with *Sclerotinia scleortiorum* fungal pathogen. Transient over-expression of miR398b in *Nicotiana benthamiana* leaves infected with *S. sclerotiorum* resulted in enhanced fungal lesions. We conclude that the miR398b-mediated up-regulation of *CSD* and *Nod19* is relevant for common bean plants to cope with oxidative stress generated in abiotic and biotic stresses.

## Introduction

The small RNAs are key post-transcriptional regulators in eukaryotes; microRNAs (miRNAs) are the best-characterized subgroup. In plants miRNAs are involved in fundamental processes such as development, phytohormonal metabolism / regulation and stress response. The plant miRNA precursors, generally transcribed by RNA Polymerase II, adopt a stem-loop structure that is excised and methylated by a dicer-like 1 (DCL1) and HEN1 proteins, respectively. Mature miRNAs are exported to the cytosol and loaded into the RNA induced silencing complex (RISC). MiRNAs repress target expression by transcript excision or translation inhibition after base complementary recognition of target mRNA transcript [1], [2].

During the recent years, the use of high-throughput sequencing technologies has facilitated the identification of miRNA populations and their target mRNAs from different plants including species from the legume family. Legumes are important for sustainable agriculture, as they are able to form nitrogen-fixing symbioses with rhizobia and soil-nutrient scavenging symbioses with mycorrhizal fungi. Common bean (*Phaseolus vulgaris*) is the most important crop legume for human consumption; it is the main source of proteins for people in countries of South-America and Africa. Upon infection with *Rhizobium etli* or *R. tropici* common bean roots develop determinate N_2_-fixing nodules [3]. Recently our groups have used different approaches to contribute to the identification and functional characterization of *P. vulgaris* miRNAs and their targets. Arenas–Huertero et al. [4] sequenced small RNA libraries and identified several conserved and six novel miRNAs, some of these responded to drought and salinity. Valdés-López et al. [5] used a macroarray-hybridization approach to identify common bean miRNAs that responded to nutrient deficiency and manganese toxicity. Targets for common bean miRNAs have been predicted through computational analyses and some conserved targets that showed a negative correlation of expression with specific miRNAs have been experimentally validated [4], [5]. The role of miR399 in the PHR1 signaling pathway for phosphorus starvation in common bean roots has been demonstrated [6]. More recently, Peláez et al. [7] used high-throughput sequencing for the identification and characterization of *P. vulgaris* miRNAs. In this work we analyzed the role of miR398 in common bean plants under abiotic and biotic stresses.

MiR398 is conserved in several plant species including legumes such as *Medicago truncatula* [8], *Lotus japonicus* [9], soybean (*Glycine max*) [10], peanut (*Arachis hypogea*) [11], urdbean (*Vigna mungo*) [12] and common bean [4], [5], [7]. Its conserved targets are transcripts coding for the Copper-Zinc Superoxide Dismutases (CSD) [13]. CSDs are scavengers of ROS and are important for stress resistance and survival in plants; they can rapidly convert highly toxic O_2_^−^ to less toxic H_2_O_2_. Besides *CSD1* and *CSD2*, other two *Arabidopsis thaliana* (Arabidopsis) transcripts coding for: Cox5b-1 (a subunit for the mitochondrial Cytochrome *c* Oxidase) and CCS1 (the Copper Chaperone for CSD) have been validated as miR398 targets [13], [14], [15]. A degradome sequencing analysis in soybean identified transcripts for *MtN19*-like (*M. truncatula* Nodulin 19-like) protein and for a serine-type endopeptidase as miR398 targets [10].

MiRNA398 was the first miRNA described as oxidative stress responsive in plants [16]. In the oxidative stress condition, generated by biotic and abiotic stresses, production of reactive oxygen species (ROS) is increased; some of these are highly toxic and must be rapidly detoxified by various cellular enzymatic and non-enzymatic mechanisms. Oxidative stress generated upon exposure to toxic concentrations of metals like copper (Cu), suppresses Arabidopsis miR398 expression that is essential for the accumulation of CSD1 and CSD2 required for ROS detoxification [16]. In addition, Arabidopsis miR398 is decreased in salt stress [17], in high light and in methyl viologen treatments [16], [18]. Down-regulation of miR398 has also been observed in *Medicago sativa* and *M. truncatula* under toxic mercury, cadmium or aluminum concentrations [19], [20]. Contrastingly, miR398 is up-regulated in nitrogen-deficient [21] and in heat-stressed Arabidopsis [22] as well as in drought-stressed *M. truncatula* [23]. In addition, miR398 responds to phosphate deficiency in different plant species such as Arabidopsis, common bean, soybean and tomato [5], [24], [25]. MiR398 is a central regulator for Cu homeostasis: its down-regulation in Cu toxicity results in high CSDs for ROS detoxification whereas in Cu deficiency increased levels of miR398 are observed together with increased Fe (iron) Superoxide Dismutase (FSD) that takes over ROS detoxification and limited Cu is delivered to Plastocyanin (PC), a Cu-containing protein that is essential for photosynthesis [15], [26]. The GTAC sequence present in the Arabidopsis miR398 promoter is an important feature in Cu responsiveness. This motif is recognized by the SPL7 transcription factor that binds to the promoter and regulates the expression of miR398. In addition SPL7 regulates the expression of other Cu-deficiency responsive miRNAs: miR397, miR408 and miR857 [27]. Moreover, Arabidopsis miR398 expression is regulated by sucrose [28]. Furthermore, the levels of miR398 decrease in Arabidopsis leaves infiltrated with avirulent strains of *Pseudomonas syringae* pv. tomato while *CSD1* was up-regulated [29].

The aim of this work was to functionally characterize miR398b in common bean plants. We confirmed the *Nod19* (*Nodulin 19*) transcript as a novel target of bean miR398. We characterized the response of miR398b and its targets *CSD1* and *Nod19* in common bean plants under Cu stress. We achieved the over-expression of miR398 in transgenic roots of bean composite plants, observing a reduction of *CSD1* and *Nod19* target gene transcripts both in control and Cu-deficiency stress conditions. In addition, the response of miR398 and its targets to symbiotic and pathogenic interactions was investigated. Our work extended the knowledge of the role of miR398b in abiotic and biotic stresses in an agronomically important legume.

## Results and Discussion

### MiR398 isoforms and target genes

The Arabidopsis miR398 family is encoded by three loci that are transcribed and processed into the miR398a, miR398b and miR398c isoforms [13], [30]. This family is highly conserved among seed plants; two and three miR398 isoforms have been detected in soybean and *M. truncatula* legume plants, respectively (www.mirbase.org). Peláez et al. [7] identified two miR398 isoforms in common bean: miR398a (20 nucleotides) and miR398b (21 nucleotides) that differ in two nucleotides and map in different loci of the *P. vulgaris* genome (www.phytozome.net, V.1.0). While miR398b was highly detected in miRNA libraries analyzed by RNA-seq, especially in seedlings and leaves, miR398a was poorly detected in all libraries [7]. In contrast to Arabidopsis miR398b and miR398c, the level of miR398a is constant in different Cu conditions tested, both in wild type and in *spl7* mutant plants lacking the SPL7 positive regulator of miR398 and Cu-responsive genes. This is consistent with the observation that the miR398a promoter does not contain GATC SPL7-DNA binding motifs [27]. Through quantitative RT-PCR (qRT-PCR) expression analysis we observed that the miR398a transcript level was very low and constant in all the tested tissues from control or Cu-stressed plants (Table S1), contrasting with our data for miR398b (see below). Therefore in this work we proceeded with the analysis of only the miR398b isoform of common bean (Phvul.008G202400.1, *P. vulgaris* genome sequence V.1.0, www.phytozome.net).

**Figure 1:**
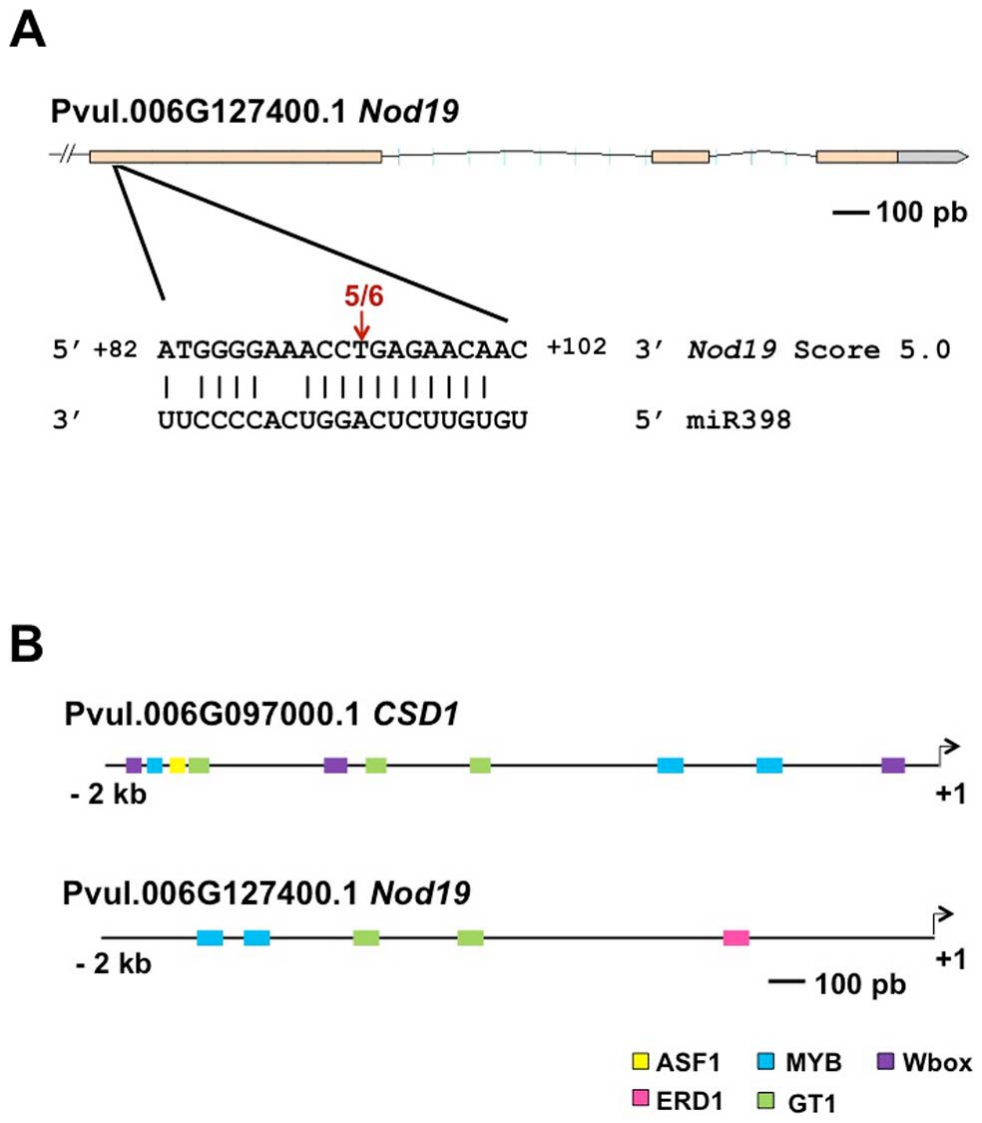
Common bean target genes for miR398. (**A**) *Nod19* gene structure according to the *P. vulgaris* genome sequence V.1.0 (www.phytozome.net). Exon regions are indicated with salmon-colored boxes and introns with black lines. The experimental validation of miR398 cleavage site was performed using a modified 5′ RLM-RACE assay. *Nod19* predicted target site is shown base-paired to miR398b; vertical lines indicate Watson-Crick base-pair and a space indicates a mismatch. The arrow above the *Nod19* mRNA indicates the number of independent clones that mapped the site of cleavage to that position. (**B**) Selected *cis*-elements identified in the promoter regions of *CSD1* and *Nod19* genes by PlantPan tool sequence analysis. Boxes for transcription factors DNA-binding are shown, these were color-coded as indicated.

Among conserved targets of miR398, *CSD1* is the most studied [16]. *P. vulgaris CSD1* gene (Phvul.006G097000.1) presents a miR398b matching site between 5′UTR and exon 1 sequence and has been validated as a miR398 target (C. De la Rosa et al., in preparation). Through a degradome study, Song et al. [10] detected the *MtN19*-like transcript (Glyma15 g13870) as a soybean miR398 target. This is orthologous to *MtN19* first identified in *M. truncatula* together with other 28 cDNA clones (nodulins *MtN1* to *MtN29*) induced during nodule development [31]. On this basis, we did a BLAST search [32] within the common bean genome sequence (http://www.phytozome.net/search.php?method=Org_Athaliana) and found Phvul.006G127300.1 locus as the *MtN19* ortholog in common bean; this could be a target for miR398b. This gene, annotated as “stress up-regulated Nod19”, is composed of three exons and 2 introns; its transcript sequence has 1418 nucleotides with 63.4% identity with *MtN19.* It encodes for a deduced 404 amino acid protein. The miR398b matching site, with a predicted score of 5.0 [13], mapped at the 5′ end of exon 1 (Fig. 1A). The 5′RLM-RACE approach was used to experimentally validate *Nod19* mRNA cleavage site. As shown in Fig. 1A, 5 out of 6 independent clones mapped the site of cleavage at the predicted position. Therefore, we demonstrated that *Nod19* is a target of common bean miR398b.

The *MtN19* gene, reported as a *M. truncatula* nodulin [31], is expressed both in young nodules and roots, but its function is still unknown [33]. *MtN19* activation is strongly reduced in the *efd-1* null deletion mutant lacking the EFD transcription factor, from the ERF family, so it has been proposed as target of this transcriptional regulator [33], [34]. *MtN19-like* genes have also been reported for other (monocot and dicot) plants such as Arabidopsis, rice, tomato, pea and *Phaseolus acutifolius* [35]. In addition to the regulation of *MtN19* during nodulation, it is induced in response to stress such as high light or drought stresses in Arabidopsis [36] and treatment with Bruchin B, a lipid-derived insect elicitor, in pea pods [35]. On this basis, it has been proposed that MtN19-like proteins that belong to the Stress Up-Regulated Nodulin 19 (SURNod19) family (Protein Families Database of Alignments and HMMS, pfam.sanger.ac.uk/) play important roles in plant stress responses [35]. To our knowledge *MtN19*-like transcripts have only been proposed as miR398 target in soybean [10], but not in *M. truncatula* or other plants. In this work we present evidence of the miR398b-mediated regulation of *Nod19*, together with *CSD1*, in common bean plants under abiotic and biotic stress conditions.

The validation of common bean *CSD1* and *Nod19* as miR398b targets supports their post-transcriptional regulation by this miRNA. However to gain insight into the transcriptional regulation of these genes we analyzed their promoter sequences (2 kb upstream from the initiation codon) with online PlantPan tool (http://plantpan.mbc.nctu.edu.tw/index.php). Figure 1B depicts selected *cis*-elements identified in the *CSD1* and *Nod19* promoters. In case of *CSD1* promoter three Wbox sites for WKRY transcription factor DNA-binding and one ASF-1 (abiotic and biotic stress differentially stimulated) site were found. ASF-1 site is activated by salicylic acid [37] while WRKY transcription factors activate transcription of specific genes mediated by this phytohormone [38]. The *Nod19* promoter contained an ERD1 (early responsive to dehydration) site. There are several GT-1 and MYB recognition sites in both promoters. The GT-1 cis-element interacts with GT-1-like transcription factor and is required for the induction of pathogen or NaCl-stress responsive genes in Arabidopsis and soybean [39]. Transcription factors from the MYB super-family are involved in different plant processes such as development, secondary metabolism and also in response to stresses such as salt and exogenous application of ABA [40]. On this basis, we can propose that, in addition to the post-transcriptional regulation by miR398, in common bean *CSD1* and *Nod19* gene expression might be regulated by stress-responsive transcription factors.

### Response of miR398b and its target genes to copper stresses

Cu^+2^ is an essential redox-active micronutrient for plant nutrition. It participates as catalytic cofactor in multiple metabolic pathways, but it can become toxic at high concentrations both for plants and animals. Plants posses several mechanisms to finely control Cu homeostasis [41].

Cu concentrations in non-contaminated soils and sediments are usually low: 20–30 ppm or<1 µM [42], [43]. Human activities such as mining, land application of sewage sludge, and discharge of untreated urban and industrial residues, led to widespread soil contamination with Cu. For example, El-Nennah et al. [44] reported 25-fold increase in Cu content in soils that had been irrigated by sewage effluents for prolonged periods (25–47 years). Cu levels in soil as high as 100-fold increased (2000 ppm) from normal levels have been recorded in mining areas and in the vicinity of Cu smelters [42]. Such high Cu concentrations are toxic and result in deleterious effects that reduce plant growth and crop productivity. Deficiency or low Cu bioavailability in soil also affects plant productivity and reduces the nutritional value of crops, thus affecting human food. For example, Cu soil concentration of 0.7 – 2.5 ppm led to abnormal growth of Douglas fir plants in the Netherlands [45].

**Figure 2:**
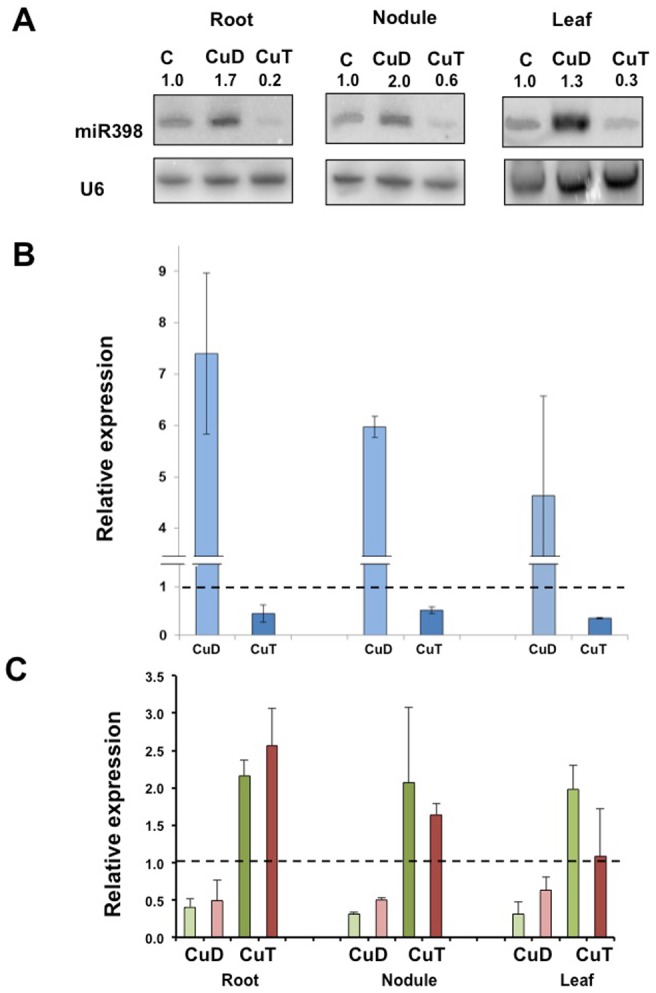
Expression pattern of miR398 and target genes *CSD1* and *Nod19* in tissues from common bean plants under copper deficiency (CuD) or copper toxicity (CuT). **(A)** miR398 levels in roots, nodules and leaves of plants grown under control (C) or stress (CuD or CuT) conditions were detected by Northern blot analysis using U6snRNA as loading control. Signal intensity of the hybridization bands was calculated and the expression ratio (stress:control) was obtained. Relative expression of **(B)** miR398b (blue) and of **(C)** target genes CSD1 (green) and Nod19 (red) in roots, nodules and leaves of plants grown under CuD (light colors) or CuT (dark colors) as determined by qRT-PCR. Values were normalized to the value from the C condition that was set to 1 as indicated with a dashed line. Values represent the average ± SD from three biological replicates.

The role of miR398 in Cu homeostasis has been previously described for Arabidopsis and other plants [16], [26]. In this work we assessed whether common bean miR398b has a similar role. We analyzed miR398b and target genes (*CSD1* and *Nod19*) expression in bean plants under Cu toxicity (CuT) or Cu deficiency (CuD) as compared to control plants growing in nutrient sufficiency. For growth of common bean plants in control and stress conditions we used a hydrponic system previously described [5]. For CuT condition the plant solution contained 70 µM CuSO_4_, equivalent to 70-fold increase as compared to the Cu level in control condition; while Cu-deprived media was used for CuD condition. Similar Cu levels have been reported for Cu toxicity studies in common bean expanding leaves or seedlings [46], [47]. The Cu-fold increase used for CuT is similar to that reported in Cu-contaminated soils [42]. The stress induced by each treatment was confirmed by the observation of characteristic visual symptoms and by the induction of marker genes that was verified in each experiment. For plants under CuT treatment the expression of the *Cytosolic Ascorbate Peroxidase* (*APX*, Phvul.011G071300) marker gene [48] was determined and *FSD* (Phvul.007G135400.1) expression [41] was determined for CuD plants. Plantlets adapted to hydroponic growth conditions were inoculated with *Rhizobium tropici,* when functional nodules were formed [5], control plants were kept growing in nutrient-full media, or changed to CuT or CuD media. After 7 days the roots, nodules and leaves from control or stressed plants were harvested for gene expression analysis (Fig. 2). We used the Northern blot approach to determine the miR398 expression in root, nodules and leaves of Cu-stressed and control bean plants. A miR398b probe was used for blot hybridization but the signals observed might reflect the combined levels of miR398b and miR398a isoforms whose sequence only differs in 2 nucleotides [7]. Similar results were obtained for the three tissues analyzed (Fig. 2A). In CuD treatment the miR398 level increased in roots, nodules and leaves as compared to levels from control plants, whereas it was almost undetectable in all the CuT-treated tissues (Fig. 2A). We used the qRT-PCR approach and a miR398b specific primer, as another, more sensitive, alternative method for the validation of the expression pattern of miR398b in control vs. Cu-stressed tissues (Fig. 2B). As compared to control conditions, in CuD the miR398b levels were increased *ca.* 7.5- 6- and 4.5-fold in root, nodules and leaves, respectively, while they were almost negligible in CuT plants (Fig. 2B). Though a similar tendency, up- or down-regulation, was observed in the two methods used, there was a variation among expression ratios (Cu-stress/control) calculated from Northern blots as compared to those from qRT-PCR analyses (Fig. 2A and B). This could be attributable to different sensitivities of the two methods and also different specificity since in Northern blot analysis we are detecting miR398a/b isoforms. The transcript levels of the *CSD1* and *Nod19* target genes in roots, nodules and leaves from control and Cu-stressed plants were determined by qRT-PCR (Fig. 2C). The expression of both target genes showed a negative correlation with miR398b expression in all the tissues and in both stress conditions (Fig. 2C). As compared to control conditions, *CSD1* and *Nod19* expression levels were decreased in CuD-stressed roots, nodules and leaves, thus indicating the miR398b-induced mRNA target cleavage (Fig. 2C). Whereas, an evident up-regulation of target genes was observed in CuT stressed tissues, except for *Nod19* in leaves (Fig. 2C).

**Figure 3:**
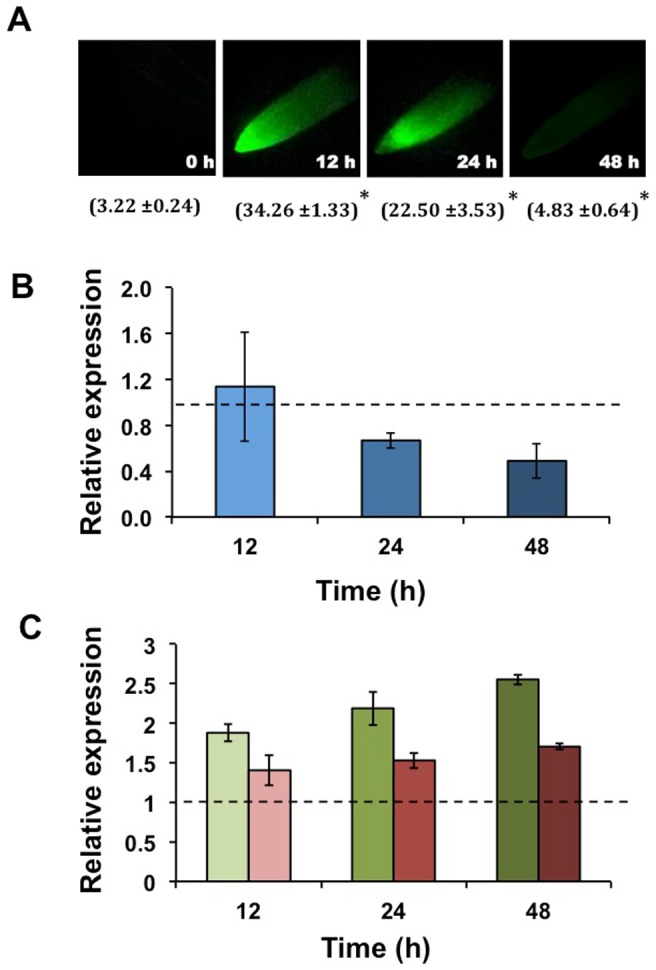
Reactive oxygen species (ROS) content and expression pattern of miR398 and target genes *CSD1* and *Nod19* in roots exposed to high Cu (CuT). Measurements were done at initial time (0 h) and after 12, 24 and 48 h of high Cu (70 µM CuSO_4_) application. (A) Histological (fluorescence) detection of ROS accumulation in CuT stressed root tips using 2′,7′- dichlorodihydrofluorescein diacetate (H_2_DCF-DA). The values in parenthesis indicate the average integrated fluorescence intensity per unit area of root tissue ±SD. Asterisk: Student's *t* test, *P*≤0.05. Relative expression, determined by qRT-PCR, of (B) miR398b (blue) and of (C) target genes *CSD1* (green) and *Nod1*9 (red) in CuT-stressed roots at the indicated time points. Values were normalized to the value from the C condition that was set to 1 as indicated with a dashed line. Values represent the average ± SD from three biological replicates.

The rapid increase in ROS concentration is called “oxidative burst”; this is better characterized when produced as a defense response to pathogen attack though it also occurs in response to abiotic stresses such as nutrient toxicity / deficiency, drought, heat stress and metal toxicity [49], [50]. Under CuT ROS are produced by autoxidation and Fenton reaction [51]. Sgherri et al. [52] reported the analysis of the early production -from 15 min to 6 h- of activated oxygen species in root apoplast of wheat following Cu excess. Also, Cuypers et al. [48] analyzed the ROS production and metabolic response of *P. vulgaris* leaves during early stages -up to 48 h- of exposure to high Cu. In this work we analyzed the response of miR398b and its target genes to the oxidative burst resulting from exposure of common bean roots to high Cu (Fig. 3). Plants were grown in hydroponic system with nutrient solution containing 70 µM CuSO_4_ and roots were harvested from 0 to 48 h after treatment. ROS content in root tips was analyzed after 2′,7′- dichlorodihydrofluorescein diacetate (H_2_DCF-DA) incubation and subsequently observed by fluorescence microscopy. A significant increase in fluorescence intensity was observed 12 h, 24 and 48 h after Cu exposure, showing a 10-fold peak at 12 h (Fig. 3A). The transcript levels of miR398b and its targets were analyzed by qRT-PCR. The level of mature miR398b showed a significant decrease at 48 h after Cu application (Fig. 3B). MiR398b differential expression inversely correlated with that of its target genes. A *ca*. 2-fold increase in *CSD1* transcript was detected after 24 h and 48 h in CuT and a minor increase (*ca.* 1.5-fold) was detected for *Nod19* transcript (Fig. 3C).

We conclude that miR398b is involved in common bean Cu homeostasis, similar to what is known for other plants [16], [26]. CuT stress is ascribed to stimulated generation of ROS that modify the antioxidant defense and elicit oxidative stress, both at late and early (oxidative burst) stages of metal exposure [48], [52], [53]. The suppression of miR398b expression in common bean roots, nodules and leaves at long period of CuT and in roots at early stages is important for the increase of *CSD1* transcript (Figs. 2 and 3) resulting in the accumulation of this enzyme important for ROS detoxification and oxidative stress tolerance. *Nod19* transcript was slightly increased mainly in roots and nodules after long Cu exposure (Fig. 2), suggesting that this protein may play a role in the oxidative stress response of common bean; however its function has not been described.

Cu is an essential element in plants, when limited in soils it provokes symptoms that affect the yield and nutritional value of crops. It participates as a redox catalytic cofactor in multiple proteins including cytochrome *c* oxidase, CSD and PC. While CSDs are dispensable for ROS detoxification since they can be replaced by FSDs, PC is essential for the photosynthetic electron flow in higher plants [26]. In Arabidopsis miR398 increases in Cu-starvation and it is involved in the mechanism to regulate Cu-containing proteins [26], [54]; our data indicate that a similar mechanism holds for Cu-deprived common bean plants. The levels of miR398 highly increased in roots, nodules and leaves of CuD bean plants lead to very low level of *CSD1* (Fig. 2) that would prioritize the delivery of limited Cu to PC.

### Over-expression of miR398 in composite plants

The study of transgenic plants with over-expression or inactivation of miRNA has allowed to gain insight or to demonstrate the regulatory functions of specific miRNAs. For example, Li et al. [55] reported the study of Arabidopsis transgenic plants over-expressing miR398b, miR160a, miR773 or miR158a that led to demonstrate the role of these miRNAs in the regulation of pathogen-associated molecular pattern-triggered plant innate immunity.

In this work we aimed to modulate the expression of miR398b to further study the role of this miRNA in common bean. In contrast to Arabidopsis, common bean and other legumes are not amenable to stable genetic transformation, and hence, protocols for high-throughput generation of transgenic legume plants are not available. The efficient protocol for *Agrobacterium rhizogenes-*mediated bean transformation to generate “composite plants” with transgenic roots and un-transformed aerial organs has been used as an alternative for stable transformation in common bean and other recalcitrant species [6], [56]. In this work we aimed to use this protocol in conjunction with constructs for over-expression or inactivation of miR398b. For miR398 inactivation we proposed to use the target mimicry strategy [57]. The MIM398 construct, with *Pv4* (*IPS1*) backbone, was designed to give rise to mimicry transcripts that specifically trap mature miR398 thus hindering miR398 activity. The OE398 construct contained the 35SCaMV promoter fused to the miR398c precursor from *M. truncatula*. Both constructs as well as the control, empty vector (EV), have the tdTomato (red fluorescent protein, RFP) reporter gene. First, the correct plant cell expression of the transgenes from each construct was tested in *Nicotiana benthamiana* leaves previously infiltrated with *Agrobacterium tumefaciens* LBA4404 bearing the corresponding plasmid. After verifying the transgene expression (data not shown) each plasmid was introduced into *A. rhizogenes* K599 and used for common bean genetic transformation and generation of composite plants [56].

A satisfactory transformation frequency (70 – 80%) was obtained with EV or OE398 plasmids. However with MIM398 plasmid, an unexpected low transformation frequency (≤20%) was obtained repeatedly in at least four independent experiments. Besides, among plants that developed hairy roots after A. *rhizogenes /* MIM398 infection only a few transgenic roots expressed RFP indicating diminished co-transformation with MIM398 binary vector. This problem was not observed for other MIM construct tested by our group at the same time nor have been reported by other groups. We hypothesize that the MIM inactivation of miR398 could affect the interaction / infection of the *A. rhizogenes* pathogen or could interfere with root development, or both (as discussed below).

We followed the analysis of common bean composite plants showing miR398b over-expression. Throughout this work we obtained several composite plants with transgenic roots bearing EV or OE398 construct. Each transgenic root results from a different transformation event and therefore each individual root may show a different degree of miR398 overexpression. Table 1 illustrates this phenomenon; the miR398 normalized expression of four representative individual OE398 transgenic roots from different composite plants varies from 3- to 9-fold as compared to expression in EV control roots. The miR398 overexpression values correlate with decreased expression of *CSD1* target gene in OE398 transgenic roots (Table 1). These results indicate that the *M. truncatula* miR398c precursor from the OE398 construct is highly transcribed and adequately processed in common bean transgenic roots. Increased transcript levels were also observed in nodules of OE398 composite plants inoculated with *R. tropici*. However, nodulation and nitrogenase activity (determined by acetylene reduction assay) was similar in OE398 and in EV inoculated composite plants thus indicating that the over-expression of miR398b had no effect in the bean-rhizobia symbiosis.

**Table 1:** Expression of miR398 and *CSD1* in individual OE398 transgenic roots.

	Expression ratio (OE398/EV)	
	miR398	*CSD1*
**HR1**	6	0.24
**HR2**	3.2	0.36
**HR3**	4.3	0.29
**HR4**	9.3	0.41

Northern blot analysis was performed using specific probes; U6 snRNA was used as loading control. Signal intensity of the hybridization bands was determined and the miR398 or *CSD1* expression ratio in OE398 as compared to control (EV) individual transgenic roots was obtained.

**Figure 4:**
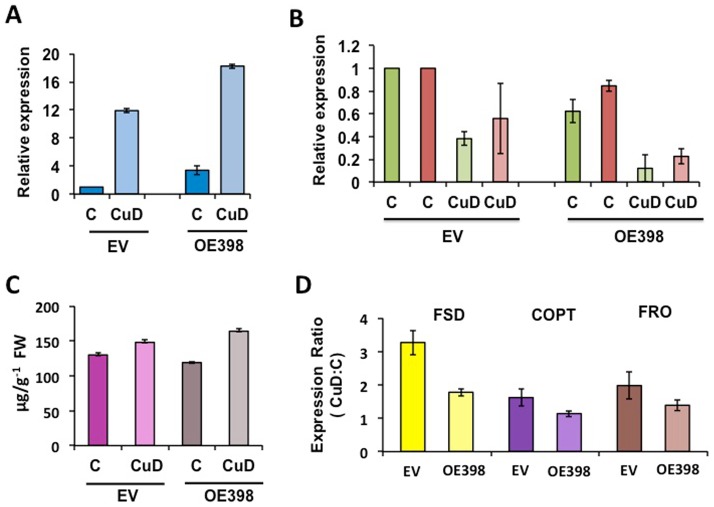
Effect of miR398b over-expression in transgenic roots from composite plants grown under CuD. Composite plants were obtained through *A. rhizogenes* transformation with EV or with OE398 plasmid, these were grown in control (sufficient nutrient) condition (C) or in CuD stress condition. (**A**) Relative expression of miR398b (blue) and of (**B**) target genes *CSD1* (green) and *Nod19* (red) determined by qRT-PCR; values were normalized to the value from the EV roots grown in the C condition that was set to 1. (**C**) Anthocyanin contents in root crown of composite plants. (**D**) Expression ratio (CuD:C) of copper-stress responsive genes: Fe superoxide dismutase (FSD, yellow), high affinity Cu transporter (COPT, purple) and ferric chelate reductase (FRO, brown). Values represent the average ± SD from three biological replicates.

We performed a comparative analysis of composite bean plants showing miR398b over-expression vs. EV composite plants grown in nutrient sufficient (control, C) or in CuD conditions, for 7 days. This experiment would allow knowing if miR398b over-expression is observed only in C growth conditions (Table 1) or also in CuD transgenic roots and if such alteration in miRNA expression would result in a much lower level of its target genes. Results are shown in Figure 4; miR398b, *CSD1* and *Nod19* transcript levels were determined by qRT-PCR from individual transgenic roots derived from biological replicates of composite plants. In C condition, the OE398 composite plant showed a 3-fold miR398 over-expression (Fig. 4A). In agreement with data presented in Fig. 2 for un-transformed plants, in CuD condition the EV composite plant showed a high endogenous miR398b induction (Fig. 4A) and a consequent decrease in *CSD1* and *Nod19* transcript levels (Fig. 4B). However the CuD-stressed OE398 composite plant showed a much higher miR398 transcript level, interpreted as the combination of over-expression and CuD response (Fig. 4A). Consequently, a very low almost undetectable level of *CSD1* and *Nod19* transcript were observed in CuD-stressed OE398 common bean (Fig. 4B). We then asked if the almost negligible level of *CSD1* and *NOD19* from OE398 transgenic roots would affect the plant response to CuD stress. For such phenotypic analysis of EV vs. OE398 transgenic roots from composite plants we determined anthocyanin content and the regulation of the expression of CuD responsive genes: *FSD*, a high affinity Cu transporters (*COPT*) and a ferric-chelate reductase (*FRO*). An increase in anthocyanin contents was observed in the crown of the root of both EV and OE398 CuD stressed plants (Fig. 4C). The accumulation of anthocyanin pigments in vegetative tissues is a hallmark of plant stress [58]. A variety of nutrient deficiencies in plants are characterized by the accumulation of flavonoids, notable red/purple colored anthocyanins, this has been well characterized in tomato leaves [59]. As mentioned before, in higher plants the abundant CSD is replaced by the Fe counterpart (FSD) upon Cu limitation, allowing plants to economize Cu when scarce, for essential chloroplastic PC [41]. In agreement, EV common bean roots from plants under CuD showed *FSD* induction (Fig. 4D). The conserved *CTR* gene family encoding high affinity Cu transporters (*COPT*) plays essential roles in Cu acquisition when this metal is limited; in Arabidopsis several members of this family (composed by 5 genes) are induced upon Cu starvation [41], [60]. A common bean *COPT* gene (Phvul.011G060400) was slightly up-regulated in EV transgenic roots subjected to CuD (Fig. 4D). The *FRO* genes encode for ferric-chelate reductase that is required in most plants to acquire Fe, by releasing Fe from organic compounds; several genes from this family are induced upon Fe limitation [61]. The enzyme encoded by Arabidopsis *FRO3* gene is involved in the reduction of divalent Cu to monovalent Cu and so, beside its regulation in Fe-deficiency, its expression is elevated in Cu-limited roots and shoots [62]. We determined the expression of a common bean *FRO* gene (Phvul.006G142300) in transgenic roots of composite plants under CuD stress and C conditions and observed an up-regulation in EV roots (Fig. 4D). In Arabidopsis induction of CuD responsive genes such as *FSD, COPT* and *FRO* as well as miR398, is positively regulated by *SPL7* that binds to GTAC motifs present in these genes' promoters [27], similar gene regulation might be occurring in common bean giving rise to the expected up-regulation response of the CuD responsive genes analyzed in EV roots under Cu deficiency (Fig 4A). Similarly, we measured the transcript level of *FSD*, *COPT* and *FRO* in OE398 roots from composite plants grown in control and CuD conditions (Fig. 4D). When comparing the CuD responsive gene expression ratios in EV vs. OE398 roots a similar trend was observed, albeit with a diminished up-regulation in OE398 composite plants indicating a decreased CuD gene response in roots with very low *CSD1* resulting from the miR398 over-expression. We suggest that the almost negligible amount of *CSD1* in CuD transgenic roots over-expressing miR398 (Figs. 4A, B) would allow to spare more Cu for its delivery to other essential Cu-containing proteins, as compared in EV roots, under Cu deficiency. In this situation the OE398 composite plants would be sensing less Cu starvation as compared to EV plants and their CuD-genes response would be diminished.

### Response of miR398 and its target genes to symbiotic and pathogenic interactions

Arabidopsis miR398 is regulated during biotic interactions with an avirulent strain of *P. syringae* pv. tomato [29]. In this work we assessed the regulation of common bean miR398b in biotic interactions, considering both a symbiont and a pathogen. It has been proposed that plant symbiosis and pathogenesis are variations on a common theme [63], [64]. The common strategies that guide the interplay between symbiotic and pathogenic plant partners include: induction of enzymes of the phenylpropanoid pathway for the synthesis of end products (flavonoids, isoflavonoids, phytoalexins) that play diverse roles in plant-biotic interactions, the hypersensitive response that entails ROS (mainly H_2_O_2_) production and induction of peroxidases as well as changes in the intracellular Ca^+2^ concentration [63], [64]. Previous works have indicated that rhizobia might be recognized as intruders that somehow evade or overcome the plant defense response [63]–[66].

#### a) Interaction with *Rhizobium tropici*

There is increasing evidence that ROS play important roles, perhaps related to signaling, in the establishment as well as in the early and later stages of the legume-rhizobia symbiosis [65], [66]. Increasing and transient ROS levels have been detected as early as seconds and up to 3 min after addition of Nod factors (NF, lipochitooligosaccharides signals secreted by rhizobia and perceived by legume roots) in common bean root hairs. This response seems to be characteristic of the symbiotic interaction since upon chitosan fungal elicitor induced a different response showing sustained increasing ROS signal [65]. In *M. truncatula* and *M. sativa* ROS production in infection threads, roots, and nodules primordia was observed from 12 to 60 h after inoculation with *Sinorhizobium meliloti* or treatment with NF [67], [68]. Accumulation of ROS in early symbiosis stages depended upon production of compatible NF by the bacteria and functional NF perception by the plant and it showed a similar pattern to the expression of an early nodulin encoding a peroxidase protein [68].

**Figure 5:**
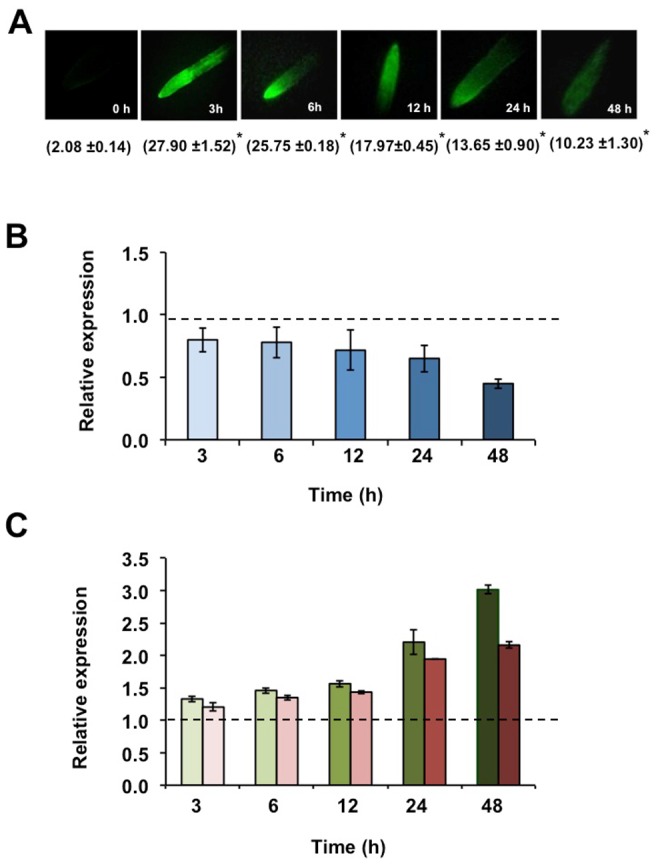
ROS content and expression pattern of miR398 and target genes *CSD1* and *Nod19* in roots inoculated with *Rhizobium tropici*. Measurements were done at initial time (0 h) and 3, 6, 12, 24 and 48 h after inoculation with *R. tropici.* (A) Histological (fluorescence) detection of ROS accumulation in inoculated root tips using 2′,7′- dichlorodihydrofluorescein diacetate (H_2_DCF-DA). The values in parenthesis indicate the average integrated fluorescence intensity per unit area of root tissue ±SD. Asterisk: Student's *t* test, *P*≤0.05. Relative expression, determined by qRT-PCR, of (B) miR398b (blue) and of (C) target genes *CSD1* (green) and *Nod1*9 (red) in inoculated roots at the indicated time points. Values were normalized to the value from the C condition that was set to 1 as indicated with a dashed line. Values represent the average ± SD from three biological replicates.

On this basis, we analyzed the regulation of miR398b and its target genes in the early stages of the common bean-rhizobia symbiosis. Plants were inoculated with R*hizobium tropici* CIAT899 and incubated in hydroponic system up to 48 h and roots were harvested at different time point to check the ROS production as well as to analyze miR398b and target genes expression (Fig. 5). Quantification of H_2_DCF-DA/ROS fluorescent complexes indicated significant ROS accumulation in roots at every time point analyzed. ROS content increased *ca.* 10-fold at 3 h to 12 h post-inoculation and it decreased at 24 h and 48 h to *ca.* 5-fold as compared to bean basal levels found in bean roots prior to rhizobia inoculation (Fig. 5A). Levels of mature miR398b decreased at early stages *R. tropici* inoculation up to *ca*. 40% at 48 h (Fig. 5B). Consequently, an increase of target genes transcripts was detected with a maximum of 3-fold for *CSD1* and 2-fold for *Nod19* at 48 h post inoculation (Fig. 5C). Results suggested that miR398b repression is important to increase *CSD1* and *Nod19* content and these could play important roles for ROS regulation in the common bean early response to rhizobia inoculation.

#### b) Interaction with Sclerotinia sclerotiorum

The plants defense response to pathogens involves rapid changes in gene, hormone and metabolite levels; miRNAs are also part of such defense mechanisms. Several miRNAs were up-regulated while others were down-regulated in Arabidopsis leaves challenged with virulent and avirulent *Pseudomonas syringae* pv. tomato strains [69]. MiR398 was the first miRNA reported to be down-regulated in response to biotic stress in Arabidopsis leaves infiltrated with avirulent strain *P. syringae* pv. tomato DC3000 [29]. In this study, *CSD1* target gene showed increased levels and therefore a negative correlation with miR398 [29]. ROS are rapidly produced in plants as a defense response to pathogen attack, a process called oxidative burst [50]. The increased CSD1 levels were likely to detoxify ROS caused by pathogen infection and support that miR398 modulated pathogen resistance in Arabidopsis. In this work we assessed miR398b regulation in common bean pathogenic interaction. This was based on Arabidopsis knowledge [29] and also in our observation of impairment of infection and hairy root formation when *A. rhizogenes* with the MIM398 construct was used. We hypothesize that such impairment in a pathogenic interaction (*A. rhizogenes –* common bean) might be related to the role of miR398 and its targets.

**Figure 6:**
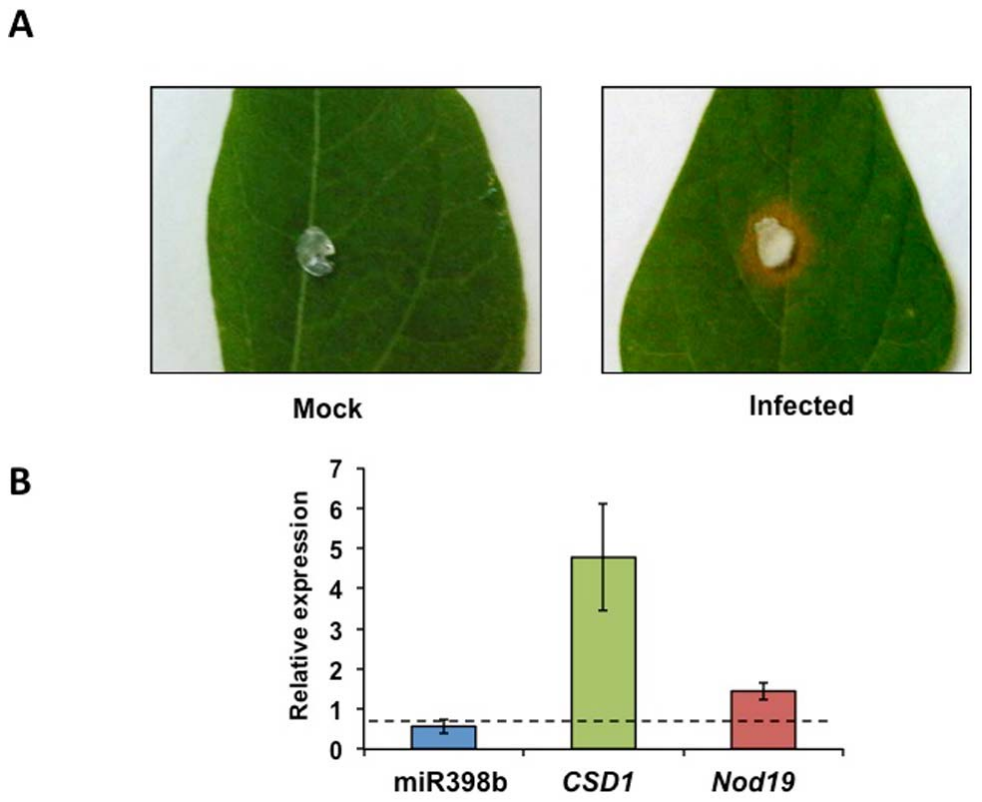
Expression pattern of miR398b and target genes *CSD1* and *Nod19* in common bean leaves infected with *Sclerotinia sclerotiorum*. (A) Mock (left) or *S. sclerotiorum* infected (right) common bean leaves after 24 h. (B) Relative expression of miR398b (blue) and of target genes *CSD1* (green) and *Nod19* (red) determined by qRT-PCR; values were normalized to the value from mock that was set to 1 as indicated with a dashed line.

*Sclerotinia sclerotiorum*, also known as white mold, is an economically important necrotrophic fungal pathogen with a broad host range [70]. *S. sclerotiorum* utilizes controlled generation of ROS for successful colonization [71], [72]. CSD, besides its role in the Cu homeostasis, plays an important role in the detoxification of ROS [49]. On this basis, we tested if common bean miR398b plays a role in *S. sclerotiorum* colonization. *P. vulgaris* is susceptible to *S. sclerotiorum* infection, the characteristic fungal lesion was clearly observed in fungi colonized leaves (Fig. 6A). The accumulation of miR398b and the expression of its two target genes (*CSD1* and *NOD19*) was evaluated by qRT-PCR in common bean leaves infected with *S. sclerotiorum*. The level of miR398b was significantly reduced in common bean leaves after 48 h of infection with *S. sclerotiorum* (Fig. 6A). In contrast, expression of *CSD1* was up-regulated in the same infected leaves (Fig. 6B). Something similar was observed in the expression of *Nod19,* however, the up-regulation of this gene upon *S. sclerotiorum* infection was lower than the induction levels observed in *CSD1* (Fig. 6B). *S. sclerotiorum* can initially suppress host oxidative burst to aid infection establishment, but later promotes ROS generation as proliferation advances [73]. The expression pattern of miR398b and its targets observed in this study (Fig. 6) might reflect part of the plant defense response against this fungal pathogen.

**Figure 7:**
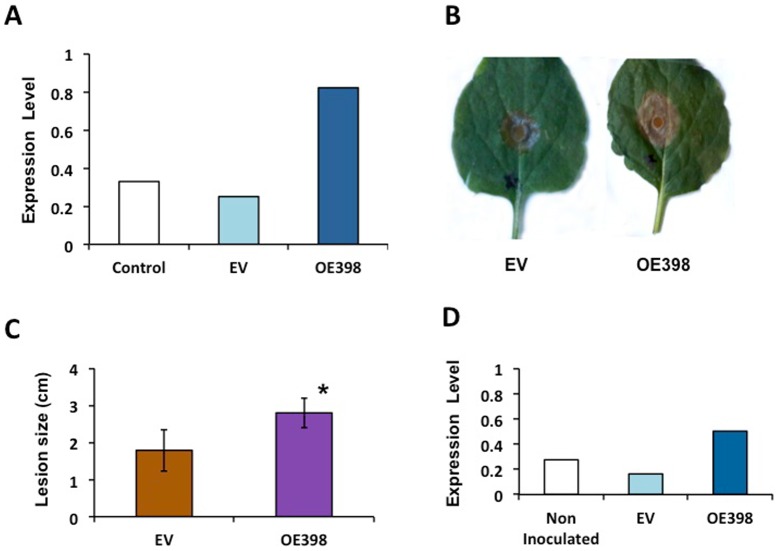
Effect of miR398b transient over-expression in *Nicotiana benthamiana* leaves infected with *Sclerotinia sclerotiorum*. *N. benthamiana* leaves were infiltrated with water (Control) or with *A. tumefaciens* bearing EV or OE398 plasmids and miR398b expression level was determined 3d after infiltration **(A)**. Subsequently, infiltrated leaves (EV or OE398) were inoculated with *S. sclerotiorum*. Characteristic fungal lesions **(B)** quantified by measuring the infection halo; asterisk: Student's *t* test, *P*≤0.01 **(C)** and miR398b expression levels determined by qRT-PCR **(D)** at 48 h after fungal infection.

Our expression analysis suggests the participation of miR398b and its targets in the infection process of *S. sclerotiorum*. In order to prove this, the precursor of miR398 was transiently over-expressed in *Nicotiana benthamiana*. Leaves infiltrated with the OE398 construct showed a 3-fold accumulation of miR398 than non-infiltrated infiltrated leaves -showing basal accumulation of endogenous *N. benthamiana* miR398- or leaves infiltrated with EV (Fig. 7A). Interestingly infiltrated leaves over-expressing miR398b were more susceptible to *S. sclerotiorum* infection which was reflected in size of lesion (Fig. 7B, C). The accumulation of miR398b remained 48 h after *S. sclerotiorum* inoculation in OE398 infiltrated leaves as compared with non-inoculated or EV inoculated leaves (Fig. 7D).

We showed that miR398 is involved in the colonization process of a symbiont (rhizobia) and of a necrotrophic pathogen. The fast and drastic increase in ROS production in common bean roots at early stages of rhizobia inoculation is in agreement with what was reported by Cárdenas et al. [65] and by Santos et al. [67] and Ramu et al. [68] for *M. truncatula* after rhizobia inoculation or NF treatment. This phenomenon has been referred to as oxidative burst, first described for pathogenic interactions and also for symbiotic interactions such as the legume-rhizobia [50], [63], [65]. We interpret that the increase in *CSD1* expression observed in the symbiotic and pathogenic common bean interactions (Figs. 5 and 6) is relevant for ROS detoxification during the oxidative burst. *Nod19* expression was also increased in these biotic interactions, though to a minor level (Figs. 5 and 7). MtN19-like from pea increases in pods treated with the insect elicitor Bruchin B [35] and thus it has been proposed that this protein from the Stress Up-Regulated Nodulin 19 (SURNod19) family plays a role in plant pathogenic and stress responses. Our finding support this proposal for common bean, though the specific function of MtN19 and orthologous proteins remain to be elucidated.

## Conclusions

In this work we performed a functional analysis of miR398b and its targets to elucidate their roles in Cu homeostasis and biotic stress in common bean.

We experimentally demonstrated *Nod19,* the common bean *MtN19* ortholog, as a miR398b target.

The role of miR398b in Cu homeostasis was evidenced through the expression analysis of this miRNA and its targets (*CSD1* and *Nod19*) in tissues of common bean plant subjected to CuT or CuD stresses. Low *CSD1*, mediated by high miR398b levels, in CuD bean tissues would spare limiting Cu for other Cu-containing proteins essential for plant processes. While high *CSD1*, correlating with miR398b down-regulation, would be relevant for detoxifying ROS produced in common bean plants under CuT. A similar response was observed in common bean during the oxidative burst generated by short-period exposure to high Cu.

High miR398b over-expression was achieved in transgenic roots from common bean composite plants that nearly lacked *CSD1* mRNA when stressed by CuD. This would provide less Cu limitation as compared to that in CuD EV composite plants that showed higher induction of CuD responsive genes (*FSD, COPT, FRO*) than OE398 plants.

We report the response of miR398b to rhizobial symbiotic and fungal pathogenic interactions. MiR398 was diminished in bean roots colonized by these microorganisms. The resulting increase in *CSD1* might be related to the oxidative burst produced in such interactions. *N. benthamiana* leaves with transient over-expression of miR398 were more susceptible to *S. sclerotiorum* infection. *Nod19* target gene expression also increased in roots colonized with rhizobia or *S. sclerotiorum*, something that might indicate its role in pathogenic interactions, though the function of Nod19 protein remains to be elucidated.

This work contributes to increase the knowledge of the roles of miRNAs in common bean, an agronomically important crop legume.

## Materials and Methods

### Plant material and growth conditions

Seeds of *Phaseolus vulgaris* Mesoamerican “Negro Jamapa 81” cultivar were surface sterilized and germinated for 2 days at 26–28 °C in darkness. Plants were grown in hydroponic system under controlled environmental conditions as previously described [5] The hydroponic trays contained 8 L of nutrient solution [74] at pH 6.5; the volume and pH were controlled along the experiment. For symbiotic conditions, N-free nutrient solution was used and 7d-old plants were inoculated with 10 mL of saturated liquid culture of *Rhizobium tropici* CIAT899. After 14d post inoculation when bean plants have developed small nodules actively fixing atmospheric N_2_, stress was applied. For Cu toxicity (CuT) the nutrient solution was supplemented with 70 µM CuSO_4_ and for Cu deficiency (CuD) the nutrient solution was deprived of Cu, for control condition the nutrient solution with 1 µM CuSO4 was maintained. Under the Cu-stress conditions used plants presented characteristic visual symptoms. In each CuT experiment the expression of *APX* (Phvul.011G071300), marker gene for CuT [48], was determined by qRT-PCR (see below). In each CuD experiment, the expression of *FSD* (Phvul.007G135400.1), marker gene for CuD [41], was determined. Increased expression of the marker genes indicated the stress-nature of the treatment used (data not shown). Roots, mature nodules or leaves from inoculated plants were harvested at 7d post stress. For analysis of roots at early stages of rhizobia infection, plants were inoculated as described and roots were collected at 0h, 3h, 6 h, 12 h, 24 h and 48 h post inoculation. For non-symbiotic conditions plants were grown in full-nutrient solution and the same CuSO_4_ concentration was used for CuT condition, roots were collected at 12 h, 24 h and 48 h.

Common bean composite plants with transgenic roots [56] were generated as described below and were grown in similar CuD or control conditions as those described for un-transformed plants. Plants were analyzed at 7d post stress. Total monomeric anthocyanin contents were measured by pH differential method using a spectrophotometer. Briefly, two different liquid extracts of the samples (crown portion of the main root) were prepared using potassium chloride buffer, pH 1.0 and sodium acetate buffer, pH 4.5 and the absorbance was measured at λ_vis-max_ and 700 nm respectively. Finally, total monomeric anthocyanin contents were determined using the absorbance values and standard formula as described before [75]. Transgenic roots were harvested for gene expression analysis through qRT-PCR.

All harvested tissue samples were immediately frozen in liquid N_2_ and preserved at -80°C until used for RNA isolation.

### ROS detection

Intracellular production of reactive oxygen species (ROS) was measured by treating the roots with 15 µM 2′,7′- dichlorodihydrofluorescein diacetate (H_2_DCF-DA) (Molecular Probes, Leiden, The Netherlands). Briefly, the roots were first washed gently with water and then left in the dye (15 µM H_2_DCF-DA) for 10–15 min under vacuumed chamber (in dark). After incubation roots were washed twice with phosphate buffer (pH 7). Fluorescence was observed at 488 nm excitation and 530 nm emission wavelengths using a fluorescence optical microscope Axioskop 2 (Zeiss). H_2_DCF-DA/ROS complexes present in the roots of bean plants were quantified based on fluorescence intensity using the NIH IMAGEJ software program (http://rsbweb.nih.gov/ij/).

### DNA sequence analysis of cis-elements

The miR398 common bean target genes considered for this analysis and their corresponding ID from the *Phaseolus vulgaris* genome sequence database available in Phytozome (www.phytozome.net, V1.0) [76], are: *Cooper/Zinc Superoxide Dismutase 1* (*CSD1*) (Phvul.006G097000.1) and *Nodulin 19* (*Nod19*) (Phvul.006G127400.1). Each downloaded promoter sequence, defined as 2 kb upstream region from the initiation codon, was inspected with Plant Promoter Analysis Navigator (PlantPAN) tool (http://plantpan.mbc.nctu.edu.tw/index.php), which identifies transcription factor binding sites in a group of gene promoters [77].

### RNA isolation

Total RNA was extracted from 1–2 g of frozen roots, leaves and nodules of bean plants using LiCl precipitation method or Trizol reagent (Invitrogen) according to the manufacturer's instruction. Integrity and quantification of RNA were checked by agarose gel electrophoresis and by absorbance measurements using a NanoDrop ND-1000 spectrophotometer (NanoDrop Technologies) respectively.

### Target validation by 5′RACE

To experimentally validate the cleavage site of Nod19 target transcript we used a modified 5′ RLM-RACE approach. Total RNA (1 µg) isolated from Cu-stressed roots was subjected to a 5′RACE reaction using FirstChoice RLM-RACE kit (Ambion) omitting calf intestine alkaline phosphatase and tobacco acid pyrophosphatase treatments. Two reverse specific primers were designed downstream of miR398 cleavage site (outer primer: 5′-GTTTCAGATCCAAGCCCAAA-3′; inner primer: 5′-GGGACACATTTTTAGGTTGG-3′). The PCR reaction and cycling conditions were setup following the manufacture's protocol. Annealing temperatures were adjusted for specific primers. Finally, the nested PCR products were cloned into pCR2.1 TOPO vector (Invitrogen) and sequenced.

### RNA gel blot analysis

Total RNA (20 µg) was separated in 15% acrylamide-7 M urea gel electrophoresis and transferred to a Hybond NX membrane (GE, Amersham) and then UV cross-linked twice. A synthetic DNA oligonucloetide with antisense sequence corresponding to miR398 (5′ CAGGGGCGACCTGAGAACACA 3′) was used as probe after labeling using [γ^32^P] ATP and T4 polynucleotide kinase (Invitrogen). As a loading control a DNA complementary to U6 snRNA (5′ CCAATTTTATCGGATGTCCCCG 3′) was used as probe. Hybridizations were performed at 42°C for 19 h in Rapid-hyb buffer (GE Healthcare). Hybridized membranes were washed twice in 2x SSC/0.1% SDS for 45 min each time and then exposed to the Phosphor Screen System (GE Healthcare). Each miRNA blot was repeated three times, representative results are shown. The intensity of each hybridization band was quantified by densitometry using the ImageQuant 5.2 software (Molecular Dynamics).

### Real-time quantitative RT-PCR (qRT-PCR)

To quantify transcript levels of mature miRNAs cDNA was synthesized from 1 µg total RNA using NCode miRNA First-Strand cDNA Synthesis kit (Invitrogen) or RevertAid H Minus First Strand cDNA Synthesis Kit (Thermo Scientific) for transcripts of target genes. Resulting cDNAs were then diluted 10-fold and used to perform the qRT-PCR experiments using SYBR Green qPCR Master Mix (Fermentas) following manufacturer's instructions. Briefly, each reaction (15 µl) contained 7.5 µl of SYBR Green, 100 nM forward primer, 100 nM universal primer and 2 µl diluted cDNA. DNase/RNase-free water was used to adjust the volume to 15 µl. The reaction mix was then incubated in a 96 well plate and analyzed using iQ5 Real-Time PCR Detection System and iQ5 Optical System Software (Bio-Rad). The thermal cycler settings were as follows: 95°C for 2 min, followed by 40 cycles of 95°C for 10 s and 55°C for 20 s. This cycle was followed by a melting curve analysis ranging from 50 to 95°C, with temperature increasing steps of 0.5°C every 10 s. Melting curves for each amplicon were observed carefully to confirm the specificity of the primers used. All qRT-PCR reactions were made by duplicate in iCycler BioRad equipment and at least two independent experiments were performed. Relative transcript levels for each sample were obtained using the ‘comparative C_t_ method’. The threshold cycle (C_t_) value obtained after each reaction was normalized to the C_t_ value of miR159 for miRNA levels or to the C_t_ value of the ubiquitin (UBC) or EF1α genes for gene levels. The expression of reference genes was constant across the conditions. The relative expression level was obtained by calibrating the ΔΔC_t_ values for the stressed conditions used and the normalized C_t_ value (ΔC_t_) for the controls. Table S2 shows the sequences of the primers used for qRT-PCR amplification of *P. vulgaris* genes. Gene models for miR398b (Phvul.008G202400.1) and *CSD1* (Phvul.006G097000), experimentally validated as miR398 target in common bean, were identified by De la Rosa et al. (in preparation). *Nod19* as well as the common bean CuD responsive genes analyzed were identified after a BLAST search [32] in the common bean genome sequence (http://www.phytozome.net/search.php?method=Org_Athaliana) based in reported gene sequences from legumes. For *Nod19* the *M. truncatula* gene sequence (*MtN19*) was used for the BLAST analysis and Phvul.006G127400.1 was identified as the ortholog gene; this gene model is annotated as “Stress up-regulated Nod19”. The common bean CuD-responsive genes identified and analyzed in this work were: *FSD* (Phvul.007G135400.1), *COPT* (Phvul.011G060400), *FRO* (Phvul.006G142300), *APX* (Phvul.011G071300). Though the *P. vulgaris* genome sequence (www.phytozome.net) gives more than one gene model for each analyzed gene, in each case we selected the one showing highest similarity with soybean orthologous genes considering that soybean has a well annotated genome sequence and it is phylogenetically close to common bean.

### Plasmid construction

To obtain a miR398 over-expression construct, initially the pTDT-DC plasmid was constructed derived from the pTDT-DC-RNAi vector [6]; it contained the 35S CaMV promoter, the attL gateway clonase reaction sites and the tdTomato gene (red-fluorescent protein, RFP) as a reporter gene. We did the construct to over-express miR398 prior to the release of the *P. vulgaris* genome sequence and so a clone from the *M. truncatula* miR398c precursor (MtrV 2Chr7_r3721) was used. Mature miRNA sequence of *M. truncatula* miR398c is identical to that of *P. vulgaris* miR398b. The *mtr-miR398c* precursor (358 bp) was cloned into the pENTR/SD/D-TOPO vector (Invitrogen) using specific forward (5′-CACCTCATTTCCATGACAACATGACA-3′) and reverse (5′-TTGTGCTTCCATCAACCAGT-3′) primers. LR clonase reaction (Gateway system, Invitrogen) between pTDT-DC and pENTR-precMiR398 provided the plasmid pOE398 to over-express miR398 under 35S promoter. To inhibit the activity of miR398c we proposed to use the artificial target mimicry strategy consisting in the expression of a modified sequence of *Pv4* (*IPS1*) containing an imperfect complementary sequence to miR398 that would reduce the miRNA-induced cleavage of its target genes [57]. The specific miR399-recognition site within *Pv4* (*IPS1*) (TC7206, Bean Gene Index DFCI) sequence was modified in vitro to obtain a mimicry sequence to miR398. We used an overlapping PCR strategy consisting in two PCR reactions: PCR1 [Pv4-Fwd (5′-CACCCAACACTCCTTCTCAAATCCTCTC-3′) + amiR398-Rev 5′- *tgtgttctcaaactgtcgcccctt*TTCAAGAGAAAATCGCC-3′] and PCR2 [amiR398-Fwd (5′- *aaggggcgacagtttgagaacaca*TTTTCCTATTCCTGGAACTCAC-3′) + Pv4-Rev 5′AGTAAGAAGCAATTTTGTTTTG 3′], the products were later mixed to obtain the Pv4 modified complete sequence. The sequence obtained was introduced into pTDT-DC vector. The empty pTDT-DC vector (used as a control, hereafter termed EV) and the resulting OE398 and pMIM398 plasmids were introduced by electroporation into *Agrobacterium rhizogenes* K599, which was then used for plant transformation.

### Fungal infection assay

Cultures of *Sclerotinia sclerotiorum* were started 48 h prior to inoculation by sub-culturing actively growing edges of fungal colonies from stock cultures onto potato dextrose agar (DIFCO). Inoculation of trifoliate leaves from young *Nicotiana benthamiana* or *P. vulgaris* plants was performed as described by Valdés-López et al. [78]. Briefly, leaves were detached and floated for 16 h in 20 ml of water in a Petri dish. Then, leaves were transferred into a Petri dish (one per trifolium) that contained moistened Whatman paper. One agar plug (4 mm diameter) with growing mycelium was placed on each leaf. Petri dishes were sealed with Parafilm and then placed in a growth chamber with controlled environmental conditions. One or two days after inoculation *P. vulgaris* or *N. benthamiana* leaves, respectively, were harvested and *S. sclerotiorum* infection levels were determined by measuring the lesion size. After this, leaves were frozen in liquid nitrogen and stored until used. Expression pattern of miR398b or target genes in fungal infected leaves was determined by qRT-PCR. The experiment was repeated three times, each at different dates and with new inoculum, to obtain three biological replicates.

### Plant transformation

For common bean transformation the protocol described by Estrada-Navarrete et al. [56] with minor modifications [79] was used to obtain composite plants with transgenic roots. Plantlets were infected with the *Agrobacterium rhizogenes* K599 strain carrying previously described constructs (EV, OE398 or MIM398). Plant growth for hairy root formation and confirmation of the expression of the reporter gene in transgenic hairy roots were done as reported [79]. Composite common bean plants carrying only fluorescent hairy roots were transferred to a hydroponic system. After 7 days of growth adaptation in hydroponics, the composite plants were transferred to control or stress treatments as described above.

For transformation of *Nicotiana benthamiana* leaves, *Agrobacterium tumefaciens* LBA4404 strain was transformed with the respective binary constructs (EV, OE398) via electroporation and grown in Luria-Broth agar / spectinomycin (100 µg/ml) plates. Just prior to the plant infiltration, a small amount of bacteria were scrapped from the plate and dissolved in 10 mM MgCl_2_. Each bacterial suspension was adjusted to OD_600_ = 0.3, and then incubated with 10 µM acetosyringone at room temperature for 2 h. Fully expanded *N. benthamiana* leaves were infiltrated by using needleless syringe. Plants were kept for three days in a growth chamber with 25°C temperature, 70% humidity and natural illumination. Leaves showing RFP fluorescence were harvested for *S. sclerotiorum* infection experiments.

## Supporting Information

Table S1 **Expression profile of miR398a.**
(DOC)

Table S2 **Primer sequences for qRT-PCR.**
(XLS)
