# Supplementary material for: Regulation of Copper Homeostasis and Biotic Interactions by MicroRNA 398b in Common Bean
Source: PLoS One. 2014 Jan 6;9(1):e84416. doi: 10.1371/journal.pone.0084416 (PMC3882225; doi:10.1371/journal.pone.0084416)
Supplement: Table S1 — Expression profile of miR398a. (DOC) [file pone.0084416.s001.doc]

**Supporting Information**

| **Conditions** | **Expression of miR398a in different tissues (dCT** ±SD**)*** | |
| --- | --- | --- |
|  | Root | Nodule |
| Control | 8.57 ±0.9 | 9.38 ±0.76 |
| CuD | 7.68 ±0.29 | 9.24 ±0.90 |
| CuT | 7.48 ±0.13 | 8.83 ±1.19 |

**Table S1. Expression profile of miR398a**

**^¶^** Average of two biological replicates and each biological replicate consists of three technical replicates

*Normalized with miR159
